# The effect of citicoline oral solution on quality of life in patients with glaucoma: the results of an international, multicenter, randomized, placebo-controlled cross-over trial

**DOI:** 10.1007/s00417-022-05947-5

**Published:** 2023-01-14

**Authors:** Luca Rossetti, Francisco Goni, Giovanni Montesano, Ingeborg Stalmans, Fotis Topouzis, Dario Romano, Eleonora Galantin, Noemi Delgado-Gonzales, Sara Giammaria, Giulia Coco, Evelien Vandewalle, Sophie Lemmens, Dimitrios Giannoulis, Theofanis Pappas, Gianluca Manni

**Affiliations:** 1 Eye Clinic, ASST Santi Paolo E Carlo, University of Milan, Via Di Rudinì, 8 20142 Milan, Italy; 2 CTIG-Teknon, Barcelona, Spain; 3 NIHR Biomedical Research Centre, Moorfields Eye Hospital NHS Foundation Trust and UCL Institute of Ophthalmology, London, UK; 4 University Hospitals Leuven, Louvain, Belgium; 5 Department of Ophthalmology, School of Medicine, Aristotle University of Thessaloniki, AHEPA Hospital, Thessaloniki, Greece; 6 IRCCS – Fondazione Bietti, Rome, Italy; 7 Eye Clinic, Policlinico Tor Vergata, Dipartimento Scienze Cliniche e Medicina Traslazionale Rome, Rome, Italy

**Keywords:** Glaucoma, Quality of life, Neuroprotection, Citicoline

## Abstract

**Purpose:**

This study aims to evaluate whether the use of citicoline oral solution could improve quality of life in patients with chronic open-angle glaucoma (OAG).

**Design:**

Randomized, double-masked, placebo-controlled, cross-over study was used. Patients were randomized to one of the two sequences: either citicoline 500 mg/day oral solution-placebo or placebo-citicoline 500 mg/day oral solution. Switch of treatments was done after 3 months; patients were then followed for other 6 months. Follow-up included 3-month, 6-month, and 9-month visits.

**Outcomes:**

The primary outcome was the mean change of “intra-patient” composite score of the Visual Function Questionnaire-25 (VFQ-25)^.^ after citicoline oral solution vs placebo at 6-month visit as compared with baseline.

**Methods:**

The trial was multicenter, conducted at 5 European Eye Clinics. OAG patients with bilateral visual field damage, a mean deviation (MD) ranging from − 5 to − 13 dB in the better eye, and controlled IOP were included. VFQ-25 and SF-36 questionnaires were administered at baseline and at 3-, 6-, and 9-month visits. A mixed effect model, with a random effect on the intercept, accounted for correlations among serial measurements on each subject.

**Results:**

The primary pre-specified outcome of the analysis reached statistical significance (*p* = 0.0413), showing greater improvement after citicoline oral solution. There was an increase in the composite score in both arms compared to baseline, but it was significant only for the placebo-citicoline arm (*p* = 0.0096, *p* = 0.0007, and *p* = 0.0006 for the three time-points compared to baseline). The effect of citicoline was stronger in patients with vision-related quality of life more affected by glaucoma at baseline.

**Conclusions:**

This is the first placebo-controlled clinical study evaluating the effect of a medical treatment aiming at improving vision-related quality of life in glaucomatous patients.

**Supplementary Information:**

The online version contains supplementary material available at 10.1007/s00417-022-05947-5.


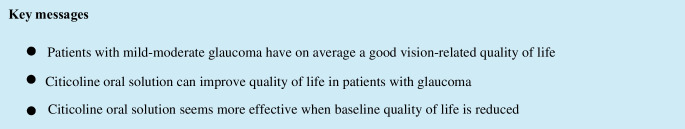


## Introduction

Open-angle glaucoma (OAG) is a progressive optic neuropathy and one major cause of global blindness [1]. The mechanisms of disease in OAG are only partially understood. Factors like elevated intraocular pressure (IOP) [1], pressure imbalance in the optic nerve head [2], impaired vascular nourishment [3], increased glial reactivity and neuroinflammation [4], and oxidative stress [5] are involved in the pathophysiology of this condition, leading to retinal ganglion cell death. As OAG shares some of these factors with other diseases showing neuronal degeneration, like Alzheimer’s or Parkinson, several authors have considered OAG a neurodegenerative disease [6].

Visual impairment is commonly associated with the disease, particularly with more advanced stages [7]. A number of reports on glaucoma patients have described difficulties in performing daily activities and loss of vision-related quality of life [8–16]. Loss of binocular visual field (VF) and central best corrected visual acuity (BCVA) have been shown to be major factors leading to reduced quality of life in glaucoma [17–19].

Treatment of the disease has the objective of slowing (or stopping) progression of damage to maintain vision-related quality of life for as long as possible [20]. Plenty of evidence clearly shows that IOP-lowering therapy can have a large effect on disease progression, thus preventing or delaying severe visual impairment [21]. Unfortunately, despite a rich armamentarium of available IOP-lowering strategies, loss of vision and even blindness are not uncommon after glaucoma even with an apparently adequate IOP control [22]. Thus, in addition to IOP-lowering treatments, complementary therapeutic strategies have also been considered in glaucoma management [6, 23].

Citicoline is a molecule that has been extensively studied in neurodegenerative diseases. A number of reports on both experimental and clinical findings have been published in the last decades on senile dementia [24, 25], stroke [26, 27], Parkinson’s disease [28] and glaucoma [29–34]. A number of studies have suggested a possible role of citicoline in the treatment of neurodegenerative diseases [35]. In particular, as an additive therapy in the treatment of glaucoma, citicoline oral solution and eye drops were found to slow down glaucoma progression in clinical trials [33, 36]. The mechanism of action of citicoline is multifarious, including the preservation of cardiolipin and sphingomyelin, restoration of phosphatidylcholine, stimulation of glutathione synthesis, lowering of glutamate concentration, rescuing mitochondrial function, and stimulating proteasome activity [6, 37]. The activities in the cholinergic and dopaminergic pathways have also been documented with a possible effect as a neuroenhancer [6]. A clinical trial on patients with stroke found that citicoline could improve cognitive status and quality of life at 2 years [38]. A systematic review of the role of citicoline as an adjunct therapy of Alzheimer’s disease showed some evidence supporting an improvement in cognition, mood, and behavioral symptoms [39].

To test the effect of citicoline oral solution on quality of life in patients with chronic OAG, we conducted a multicenter, randomized, placebo-controlled cross-over study.

## Methods

The trial was conducted at 5 University Eye Clinics in Rome, Barcelona, Leuven, Thessaloniki, and Milan between winter 2019 and summer 2021. The study was designed following the tenets of the Declaration of Helsinki, and the protocol was submitted and approved by each University Ethics Committee. The trial was funded by Omikron Italia®a srl and registered (EudraCT 2018–002187-11, clinicaltrials.gov).

### Patients in the trial

Glaucoma definition was based on VF damage (24–2 SITA standard strategy, Humphrey Visual Field Analyser, HFA) corresponding to glaucomatous changes at the optic nerve head (ONH) irrespective of IOP. Inclusion criteria were patients with OAG, including pseudoexfoliative and pigmentary glaucomas; age ≥ 18 years; presence of bilateral visual field damage; a level of moderate damage in the better eye, with a mean deviation (MD) ranging from − 5 to − 13 dB at the screening assessment; controlled IOP (according to physician’s judgement); and a signed consent form to participate in the study. Exclusion criteria were single-eyed patients; patients without those psychophysical requirements to adequately participate and complete the trial; patients with other types of glaucoma; patients with other ocular comorbidities interfering with the correct assessment of the glaucomatous damage to the VF; patients who had undergone surgery within 6 months; patients taking other potential neuroprotectors; patients with Parkinson’s disease, dementia or a diagnosis of stroke in the last 6 months.

### Study design

The trial was a randomized, double-masked, placebo-controlled, cross-over study. Patients who accepted to participate in the trial signed an informed consent form and were randomized to one of the two sequences of the cross-over design: either citicoline 50 mg/ml oral solution-placebo or placebo-citicoline 50 mg/ml oral solution. The randomization was stratified by center.

The study protocol included the following visits:

1. Baseline visit (beginning of 1st period): Patients underwent a complete ophthalmic examination, including BCVA, biomicroscopy (with a specific lens evaluation using the Lens Opacities Classification System III, LOCS III, criteria [40]) IOP measurement, fundus evaluation, and gonioscopy. A VF test (24–2, SITA standard strategy, HFA) was also performed. A trained evaluator masked to treatment administered the two study questionnaires (VFQ-25 and SF-36). Finally, those patients entering the study received citicoline 50 mg/ml oral solution or placebo bottles, randomly assigned, for the first 3-month period.
2. 3-month visit (end of the 1^st^ period and beginning of 2^nd^ period): Patients were asked about treatment side effects, and all complains/considerations were recorded. The two study questionnaires (VFQ-25 and SF-36) were then administered. A complete ophthalmic examination and a VF test with the same strategy were performed. Patients received the assigned bottles for the second 3-month period.
3. 6-month visit: Patients were asked again about treatment side effects, and all complains/considerations were recorded. Patients were again administered the 2 study questionnaires (SF-36 and VFQ-25). A complete ophthalmic examination and a VF test with the same strategy were performed. Patients received the assigned bottles for the third 3-month phase.
4. 9-month visit (end of the 2st period): Patients were asked again about treatment side effects and administered the two study questionnaires (VFQ-25 and SF-36). A final, complete ophthalmic examination and a VF test with the same strategy were performed.

### Study treatments

Patients were treated with any IOP-lowering agent to control the disease. Patients were randomized to a citicoline 50 mg/ml oral solution-placebo or placebo-citicoline 50 mg/ml oral solution sequence and received treatment for 3 months in the first period and for 6 months in the second period of the cross-over design. The second period was extended to 6 months to control for a potential carry-over effect in the group receiving citicoline oral solution in the first period of the cross-over design.

Two identical bottles contained either 500 ml of citicoline oral solution (Neurotidine®, Omikron Italia srl), citicoline free acid 50 mg/ml; water; fructose; acidity regulators: sodium citrate, sodium hydroxide; preservative: potassium sorbate; color: riboflavin; or placebo 500 ml oral solution, water; fructose; sucralose; acidity regulators: sodium citrate, anhydrous citric acid, sodium hydroxide; preservative: potassium sorbate; color: riboflavin. Citicoline oral solution or placebo were administered at a dosage of 10 ml (500 mg of citicoline/day) in the morning.

### Study outcomes

The primary outcome was the mean change of “intra-patient” global score of the VFQ-25 questionnaire after citicoline oral solution vs placebo at 6 months as compared with baseline. Secondary outcomes were the difference in the change from baseline at 3 and 9 months; the comparison between different time-points in each arm with the respective baseline; the comparison between the two arms at each time-point of the two study questionnaires, VFQ-25 and SF-36, and the safety and tolerability of citicoline oral solution.

### Analysis

The trial sample size was calculated on the main outcome of the study. With a sample size in each sequence group of 100 (a total sample size of 200), a 2 × 2 crossover design would have 80% power to detect a difference in mean intra-patients global score of 3.0 assuming that the crossover ANOVA √MSE is 10.607 (the standard deviation of differences, σd, is 15.0) with a 0.05 two-sided significance level. The total sample size was adjusted to 220 patients considering an expected dropout rate of about 10%.

The primary outcome analysis was performed on the composite score of the Visual Function Questionnaire-25 (VFQ-25) [41], calculated according to the published manual. A mixed effect model, with a random effect on the intercept, accounted for correlations among serial measurements on each subject. The fixed effects were the sequence (randomization arm) and the time-points (baseline, month 3, month 6, and month 9). Interactions between the fixed effects allowed for non-monotonic trends and captured the effect of the change in treatment between the two arms. For the analysis by treatment, the sequence in the model for the main outcome was replaced by a categorical factor indicating the treatment received at each time point. This factor had three levels (“no treatment,” “placebo,” and “citicoline oral solution”). The model was then used to perform pair-wise comparisons between the effect of the three levels (Bonferroni-Holm correction for 3 comparisons). The interaction term modelled the carry-over effect, indicating a different effect of each treatment for each time-point. All calculations were performed in R (R Foundation for Statistical Computing) using the *lme4* package [42] and the *lsmeans* package [43]. All study centers filled in a web-based e-CRF. The Data Center checked the e-CRF and solved all the queries.

## Results

**Fig. 1:**
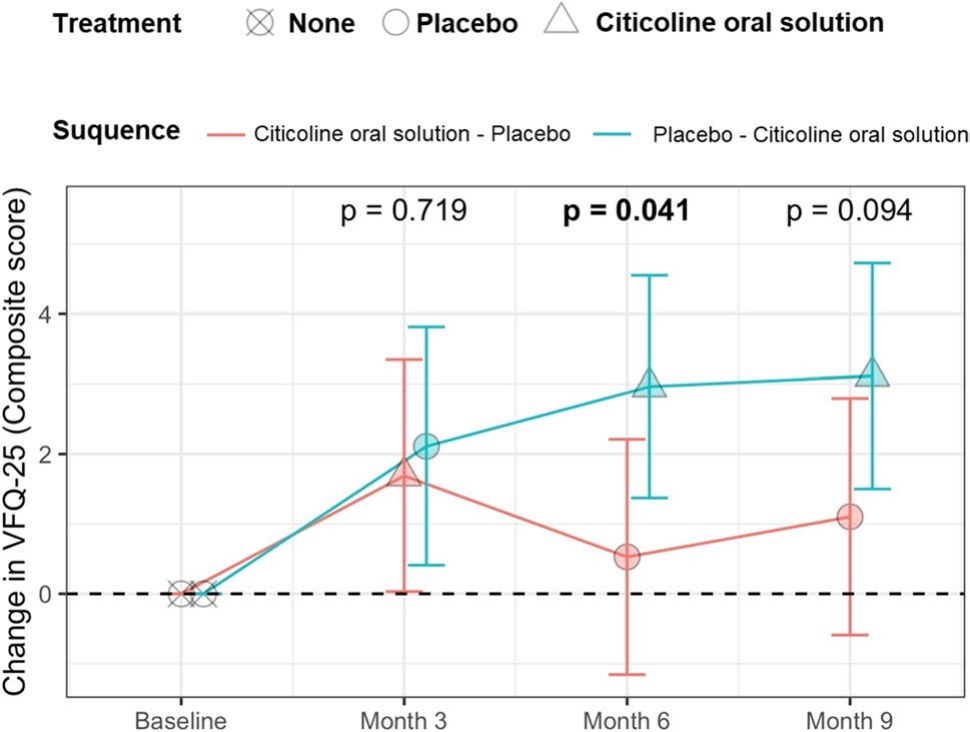
Graphical representation of the results in Table 2 (change from baseline). The dots represent the estimates from the model; the error bars represent the 95% confidence intervals

**Fig. 2:**
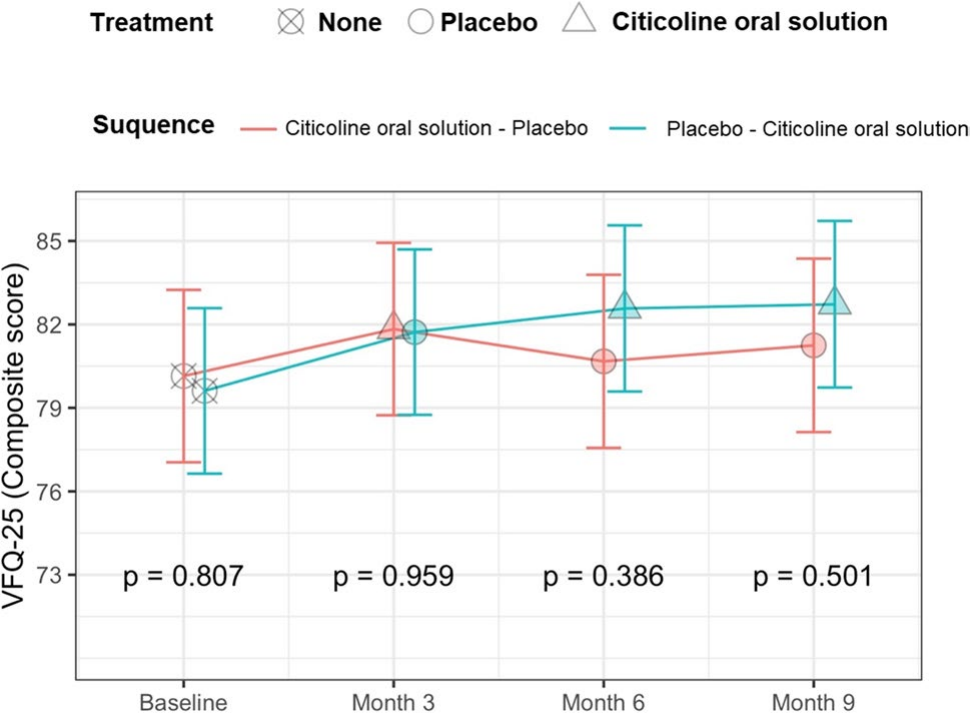
Graphical representation of the results in Table 2 (composite scores). The dots represent the estimates from the model; the error bars represent the 95% confidence intervals

One-hundred-fifty-five patients were included in the study. Four patients were excluded because not properly randomised. Four patients from the citicoline oral solution-placebo arm and one from the placebo-citicoline oral solution arm were excluded because they did not complete any visit beyond the baseline, as reported in the CONSORT flow diagram (Supplementary Fig. 1). Table 1 presents the patients’ main characteristics. The estimates of the model for the primary outcome are reported in Fig. 1 and in Table 2. Citicoline oral solution provided a significant improvement from the baseline score at 6 months as compared to placebo (*p* = 0.0413). There was an increase in the composite score in both arms compared to baseline, but it was significant only for the placebo-citicoline oral solution arm (*p* = 0.0096, *p* = 0.0007, and *p* = 0.0006 for the three time-points compared to baseline). Of note, patients who used citicoline oral solution at the first time-point showed a reduction in the composite score when switched to placebo, but this effect did not reach statistical significance (*p* = 0.1770). When compared directly, there was no significant difference in the composite score between the two arms at any time point (Fig. 2).

**Table 1:** Descriptive statistics at baseline, reported as median [interquartile range] for continuous values. BCVA = best corrected visual acuity; MD = mean deviation; RNFL = retinal nerve fiber layer; VFQ = Visual Function Questionnaire; SF = Short Form health survey. RNFL thickness and intraocular pressure are for the better eye. *One female subject missing

	Citicoline oral solution–placebo (*N* = 70)	Placebo-citicoline oral solution (*N* = 76)
Age (years)	71 [63, 78]	69 [61, 75]
Sex (male/female)	31/39	42/34
Better BCVA (decimals)	0.9 [0.8, 1.0]	1.0 [0.8, 1.0]
Better MD (dB)	− 8.03 [− 10.33, − 6.52]	− 8.84 [− 10.21, − 7.08]
Average RNFL thickness (µm)	61.50 [55.50, 70.00]	61.75 [53.38, 69.12]
Intraocular pressure (mmHg)	13 [12, 15]	13 [11, 15]
Baseline VFQ-25 (composite)	82.16 [72.88, 92.16]	80.93 [73.84, 89.32]
Baseline SF-36 (mental health)	51.24 [45.27, 54.93]	50.54 [44.44, 55.33]*
Baseline SF-36 (general health)	51.40 [47.30, 56.17]	51.58 [42.18, 56.95]*

**Table 2:** Results of the sequence analysis at different time points. The table reports the estimate [95% confidence intervals] of the composite score from the model. The *p*-value are for the comparisons between the two arms at each time point

	Time point	Citicoline oral solution-placebo	Placebo-citicoline oral solution	*p*-value
Change from baseline	Month 3	1.69 [0.03, 3.34]	2.11 [0.41, 3.81]	0.7190
	Month 6	0.53 [− 1.15, 2.21]	2.96 [1.37, 4.55]	**0.0413**
	Month 9	1.1 [− 0.59, 2.79]	3.11 [1.5, 4.73]	0.0940
Composite score	Baseline	80.15 [77.05, 83.24]	79.61 [76.64, 82.59]	0.8070
	Month 3	81.83 [78.74, 84.93]	81.72 [78.75, 84.70]	0.9594
	Month 6	80.67 [77.56, 83.79]	82.57 [79.59, 85.56]	0.3857
	Month 9	81.25 [78.13, 84.37]	82.73 [79.73, 85.72]	0.5010

Bold: *p*-value <0.05

**Fig. 3:**
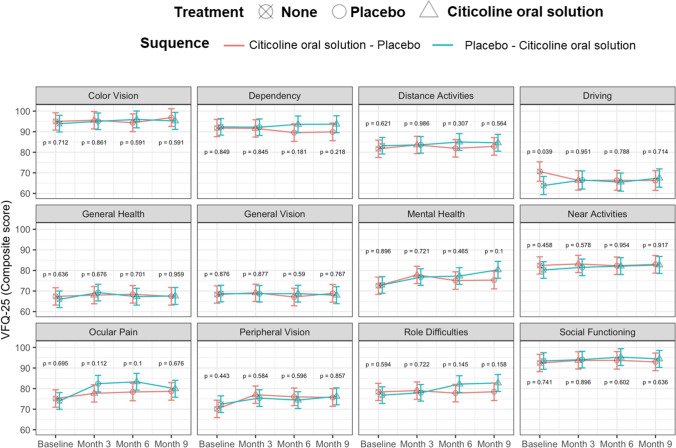
Sequence analysis for each subscale in the VFQ-25 questionnaire. The dots represent the estimates from the model; the error bars represent the 95% confidence intervals

The results of subscale score analysis are reported in Fig. 3. There were no significant differences between the two arms at any time-point. The only significant difference in change from baseline was found for “driving” at 9 months (*p* = 0.0334).

Overall, both placebo and citicoline oral solution had a significant effect compared to no treatment (i.e., to baseline, see Fig. 3; Table 3). Citicoline oral solution provided a small improvement on the composite score over the placebo, with borderline significance (*p* = 0.0459; see Table 3). The carryover effect was tested and was not statistically significant (*p* = 0.9738). Of all the subscales, the only significant difference between citicoline oral solution and placebo was found for role difficulties (*p* = 0.0349).

**Table 3:** Results of the treatment analysis. The table reports the estimate [95% confidence intervals] of the composite score from the model. The *p*-value are for the pairwise comparisons between different treatment (None = baseline). Bonferroni-Holm correction for three tests

	None	Citicoline oral solution	Placebo	None-citicoline oral solution	None-placebo	Citicoline oral solution-placebo
Composite	79.87 [77.73, 82.01]	82.32 [80.22, 84.41]	81.30 [79.20, 83.40]	** < 0.0001**	**0.0175**	**0.0459**
Color vision	94.43 [91.52, 97.34]	95.54 [92.90, 98.17]	95.48 [92.82, 98.13]	1.0000	1.0000	1.0000
Dependency	92.08 [89.18, 94.98]	92.89 [90.26, 95.52]	90.70 [88.05, 93.34]	0.6852	0.6852	0.2868
Distance activities	82.47 [79.57, 85.38]	84.33 [81.70, 86.97]	82.91 [80.26, 85.55]	0.5984	0.7674	0.5984
Driving	66.94 [63.72, 70.15]	66.35 [63.51, 69.19]	66.45 [63.56, 69.35]	1.0000	1.0000	1.0000
General health	66.66 [63.76, 69.56]	67.54 [64.91, 70.17]	68.47 [65.83, 71.12]	0.9596	0.6401	0.9596
General vision	68.53 [65.62, 71.43]	68.55 [65.92, 71.18]	68.31 [65.66, 70.95]	1.0000	1.0000	1.0000
Mental health	72.77 [69.87, 75.68]	78.40 [75.77, 81.03]	75.84 [73.20, 78.49]	0.0003	0.0704	0.0704
Near activities	81.29 [78.38, 84.19]	82.57 [79.94, 85.21]	82.29 [79.65, 84.94]	1.0000	1.0000	1.0000
Ocular pain	74.57 [71.67, 77.47]	80.28 [77.65, 82.91]	79.99 [77.35, 82.64]	**0.0002**	**0.0004**	0.8293
Peripheral vision	71.33 [68.41, 74.25]	75.80 [73.16, 78.43]	75.75 [73.09, 78.41]	**0.0067**	**0.0067**	0.9719
Role difficulties	77.57 [74.67, 80.47]	81.27 [78.64, 83.90]	78.14 [75.49, 80.78]	**0.0320**	0.6956	**0.0349**
Social functioning	92.95 [90.05, 95.85]	94.41 [91.78, 97.04]	93.69 [91.04, 96.33]	0.9404	1.0000	1.0000

Bold: *p*-value <0.05

**Fig. 4:**
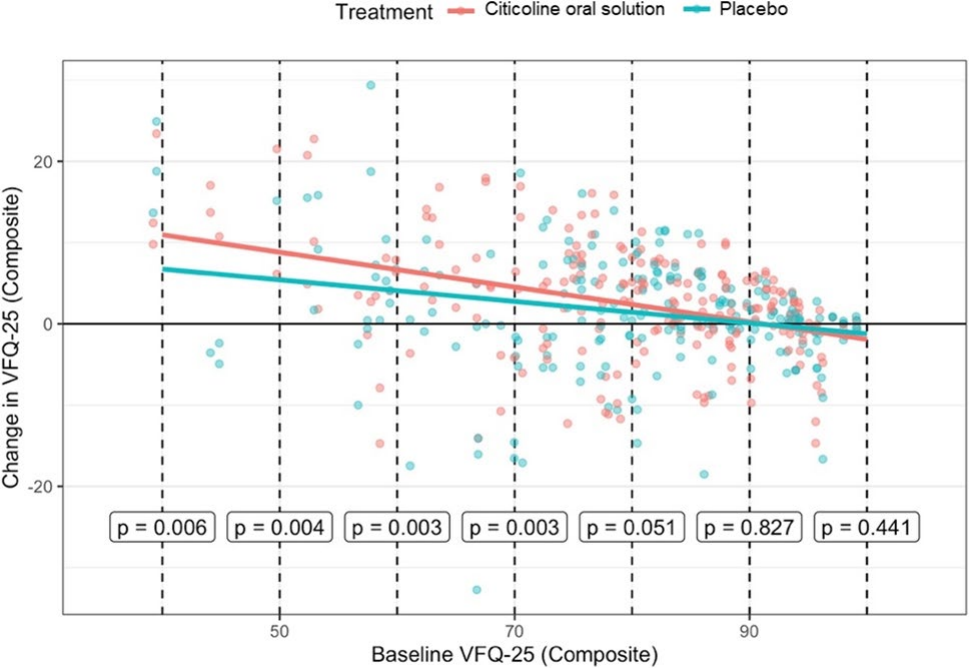
Change from baseline obtained with citicoline oral solution and placebo stratified by baseline composite score

A mixed effect model was used to explore the effect of the baseline composite score on the change obtained with the placebo and citicoline oral solution respectively. The differential effect of the treatment was modelled through an interaction term between the treatment and the baseline composite score. If significant, this indicates a significant difference in the effect of citicoline oral solution compared to placebo at the same baseline score. The *p*-value for this interaction term was *p* = 0.0248 (Fig. 4). Most of sample had a baseline close to the ceiling of the measurement (i.e., scores close to 100), indicating that an improvement could be effectively measured for a minority of the sample.

A model similar to the one for the baseline score was used to study the correlation between the mean deviation (MD) of the better eye and the composite score. MD had a significant effect on the composite score (*p* = 0.0081 for the slope, but there was no significant difference in slopes between citicoline oral solution and placebo (*p* = 0.4635). BCVA had a significant effect on the composite score (*p* < 0.0001 for the slope), but there was no significant difference in slopes between citicoline oral solution and placebo (*p* = 0.260).

The analysis of SF-36 questionnaire was conducted using the same approach as the VFQ-25. The only difference was that the two main summary scales, the mental component, and physical component summary, needed to be kept as separate scores. The estimates of the model for the main outcome are reported in Table 4. The only significant difference was for the mental health score at baseline, therefore independent of the treatment. There was no significant change between time-points in either sequence (smallest *p* = 0.7656). There was no difference between the two arms at 6 months (*p* = 0.2234 and *p* = 0.7440 for the mental and physical component summary, respectively). Of all the subscales, the only significant difference was found between citicoline oral solution and baseline for social functioning (*p* = 0.0251, Bonferroni-Holm correction for 3 comparisons; see Table 5). The MD had no significant effect on the scores (global *p* = 0.7186 for the slopes). The BCVA had a significant effect on the general health summary score (*p* = 0.00936 for the slope) but not of the mental health summary score (*p* = 0.5574). There was no significant difference in slopes between citicoline oral solution and placebo (global *p* = 0.5909).

**Table 4:** Results of the sequence analysis at different time points. The table reports the estimate [95% confidence intervals] of the composite score from the model. The *p*-value are for the comparisons between the two arms at each time point

	Time point	Citicoline oral solution-placebo	Placebo-cicitoline oral solution	*p*-value
Mental component summary	Baseline	51.24 [49.12, 53.35]	48.22 [46.18, 50.26]	**0.0443**
	Month 3	50.86 [48.76, 52.96]	49.03 [47.01, 51.05]	0.2184
	Month 6	50.99 [48.86, 53.12]	50.07 [48.00, 52.15]	0.5447
	Month 9	49.75 [47.59, 51.91]	50.04 [47.91, 52.17]	0.8508
Physical component summary	Baseline	48.96 [46.84, 51.07]	49.60 [47.56, 51.64]	0.6689
	Month 3	50.15 [48.04, 52.25]	49.95 [47.93, 51.97]	0.8973
	Month 6	49.80 [47.67, 51.92]	49.87 [47.80, 51.95]	0.9592
	Month 9	49.58 [47.41, 51.74]	49.77 [47.64, 51.91]	0.9000

**Table 5:** Results of the treatment analysis. The table reports the estimate [95% confidence intervals] of the composite score from the model. The *p*-value are for the pairwise comparisons between different treatment (None = baseline). Bonferroni-Holm correction for three tests

	None	Citicoline oral solution	Placebo	None-citicoline oral solution	None-placebo	Citicoline oral solution-placebo
Mental component summary	49.68 [48.21, 51.14]	50.41 [49.09, 51.73]	49.80 [48.49, 51.12]	1.0000	1.0000	1.0000
Physical component summary	49.29 [47.82, 50.76]	50.02 [48.70, 51.34]	49.70 [48.38, 51.02]	1.0000	1.0000	1.0000
Bodily pain	69.29 [65.75, 72.84]	71.47 [68.25, 74.70]	71.65 [68.43, 74.87]	0.5378	0.5378	0.9124
General health	63.88 [60.34, 67.42]	62.91 [59.68, 66.13]	62.45 [59.23, 65.67]	1.0000	1.0000	1.0000
Mental health	70.45 [66.91, 73.99]	71.31 [68.08, 74.54]	71.28 [68.06, 74.50]	1.0000	1.0000	1.0000
Physical functioning	75.81 [72.27, 79.35]	77.98 [74.75, 81.20]	75.76 [72.54, 78.98]	0.4925	0.9805	0.4925
Role-physical	79.06 [75.52, 82.60]	82.27 [79.05, 85.50]	81.08 [77.86, 84.30]	0.2024	0.4976	0.4976
Role-emotional	84.79 [81.25, 88.33]	86.24 [83.02, 89.47]	83.05 [79.84, 86.27]	0.6460	0.6460	0.1349
Social functioning	77.98 [74.44, 81.52]	82.62 [79.39, 85.84]	80.59 [77.37, 83.81]	**0.0251**	0.2736	0.2736
Vitality	60.85 [57.31, 64.39]	61.53 [58.31, 64.76]	61.75 [58.53, 64.97]	1.0000	1.0000	1.0000

Bold: *p*-value <0.05

## Discussion

This is the first placebo-controlled clinical study evaluating the effect of a medical treatment aiming at improving vision-related QoL in glaucomatous patients. The primary pre-specified outcome of the analysis (difference in the change from baseline at six months) reached statistical significance (*p* = 0.0413), showing greater improvement after citicoline oral solution. A significant effect was also found between citicoline oral solution and placebo on the VFQ-25 composite score when comparing the composite score for the VFQ-25 by pooling the effect of treatment across time points. The effect was stronger when analyzing the patients who had their vision-related quality of life more affected by glaucoma at baseline. In fact, the improvement under treatment was proportional to the baseline VFQ-25 composite score, for both citicoline oral solution and placebo. The slope of this relationship was however significantly steeper for citicoline oral solution, indicating larger improvements for lower starting scores compared to placebo. The effect of baseline also explains why a significant change from baseline could be observed at six months, despite no statistically significant difference in the actual composite score between the two arms. Both the placebo and citicoline oral solution showed a positive effect on the vision related quality of life. Interestingly, the composite score kept increasing, on average, for patients who switched from placebo to citicoline oral solution and generally dropped for patients switching from citicoline oral solution to placebo. The VFQ-25 appeared to appropriately quantify vision related quality of life and was in fact significantly correlated to the MD and the BCVA of the better eye. The cross-over design was primarily meant for analysis comparing intra-patient change (primary outcome) from baseline with 6-month time-points. The 9-month extension was introduced to assess potential “carry-over” effects at 6 months. We did not find any significant differences between the 6-month and the 9-month time-points.

Citicoline oral solution did not show any significant effect on the general quality of life (SF-36) which also showed poor correlations with metrics of visual function. The fact that only the visual questionnaire (VFQ-25) and not the general health questionnaire (SF-36) showed a significant improvement suggests that the citicoline effect was more related to visual function than just to the general health status in our study involving glaucoma patients.

There is a copious literature supporting citicoline effect in glaucoma and more in general in neurodegenerative diseases [6, 24–39]. Such an effect was clearly demonstrated in experimental studies [37, 44–46] and in clinical settings as well [24–39]. Trials on glaucoma patients showed a beneficial role of citicoline in improving electrophysiology [29–32] and a possible effect on visual field changes [36]. In a recently published clinical trial, citicoline (administered as eyedrops) was found to be associated with a reduction of glaucoma progression in patients with apparently controlled IOP [36]. All these encouraging data led to the assumption that citicoline might have a neuroprotective effect as described above. Clinical data on a number of neurodegenerative diseases like Parkinson’s disease, senile and vascular dementia, and stroke seem to confirm experimental observations [35]. Several studies have investigated the effect of citicoline on vision [6, 29–36]. This action would be probably mediated by the stimulation of the dopaminergic system in the visual pathways: citicoline was found to improve visual acuity, visual evoked responses, and contrast sensitivity in glaucoma [29–32], amblyopia [47–49], and in non-arteritic ischemic optic neuropathy [50, 51].

The findings of our trial confirm the effect of citicoline as a neuroenhancer as the short duration of the study did not allow to show any significant disease change. Data on visual field confirm that no progression occurred in the 9-month follow-up time. The “good IOP control” (i.e., 13 mmHg on average in both groups) reinforces the likelihood of a lack of progression in these patients. In fact, differently from what could be observed in another trial, this sample was not selected based on glaucoma progression despite IOP control [36]. The effect of citicoline decreased after the switch to placebo. This finding seems to confirm the observations of Parisi et al. who could show an improvement in the electrophysiological function of the retina in glaucoma patients with an effect clearly measurable after 4 months of treatment that, however, regressed to normality after citicoline was stopped. The authors tried to interpret their findings suggesting a neuro-enhancer action of the molecule [32].

Among study limitations, it is worth mentioning the reduced sample size of the study. Of the 220 patients that were planned, only 155 were finally enrolled. The drop-out rate was low (5%), less than predicted, despite the trial period fell completely into “pandemic time.” Average values of composite score in the 2 arms showed that overall quality of life was rather good, similar to the one observed in other clinical trials including patients with moderate glaucoma. It is possible that the reduced statistical power of the study did not allow to find differences in secondary outcomes between citicoline and placebo; and the same comment holds for subscales analyses. Another possible limitation is the lack a of wash-out period between the 2 cross-over phases. We do not think it has had a meaningful impact on our results, as confirmed by the lack of significant differences between the 6-month and 9-month time-points. However, any potential carry-over effect would have acted to dilute the difference between the two arms after the switch, potentially reducing the chances of detecting a significant change from baseline, but this was not the case.

To conclude, this trial supports the effect of citicoline oral solution on improving the vision related quality of life, measured by the VFQ-25, with no positive or negative impact on the general quality of life, measured by the SF-36. The VFQ-25 composite score at baseline was generally high, making harder to show an impact of glaucoma on quality of life in the study population. Future investigations should focus on the recruitment of participants with more advanced bilateral VF damage, in whom a compromised quality of life can be more likely observed.

## Supplementary Information

Below is the link to the electronic supplementary material.

Supplementary file1 (PDF 208 KB)
