# Supplementary material for: The effect of citicoline oral solution on quality of life in patients with glaucoma: the results of an international, multicenter, randomized, placebo-controlled cross-over trial
Source: Graefes Arch Clin Exp Ophthalmol. 2023 Jan 14;261(6):1659–68. doi: 10.1007/s00417-022-05947-5 (PMC10199108; doi:10.1007/s00417-022-05947-5)

The effect of citicoline oral solution on quality of life in patients with glaucoma. The results of an international, multicenter, randomized, placebo-controlled cross-over trial

Luca Rossetti, MD[1], Francisco Goni, MD[2], Giovanni Montesano, MD[3], Ingeborg Stalmans, MD[4], Fotis Topouzis, MD[5], Dario Romano, MD[1], Eleonora Galantin, MD[1], Noemi Delgado-Gonzales, OD[2], Sara Giammaria, MD[6], Giulia Coco, MD[7], Evelien Vandewalle, MD[4], Sophie Lemmens, MD[4], Dimitrios Giannoulis, MD[5], Theofanis Pappas, MD[5], Gianluca Manni, MD[7]

1 Eye Clinic, ASST Santi Paolo e Carlo, University of Milan, Italy
2 CTIG-Teknon, Barcelona, Spain
3 NIHR Biomedical Research Centre, Moorfields Eye Hospital NHS Foundation Trust and UCL Institute of Ophthalmology, London, UK
4. University Hospitals Leuven, Belgium
5. Department of Ophthalmology, School of Medicine, Aristotle University of Thessaloniki, AHEPA Hospital, Greece
6. Bietti Foundation, Rome, Italy
7. Eye Clinic, Policlinico Tor Vergata, Dipartimento Scienze Cliniche e Medicina Traslazionale Rome, Italy

Corresponding author: Luca Rossetti, Clinica Oculistica, ASST Santi Paolo e Carlo Via di Rudinì, 8 20142 Milano, Italy, luca.rossetti@unimi.it

Supplementary Fig. 1 CONSORT flow diagram showing enrollment, allocation, follow-up and analysis of patients involved in the trial

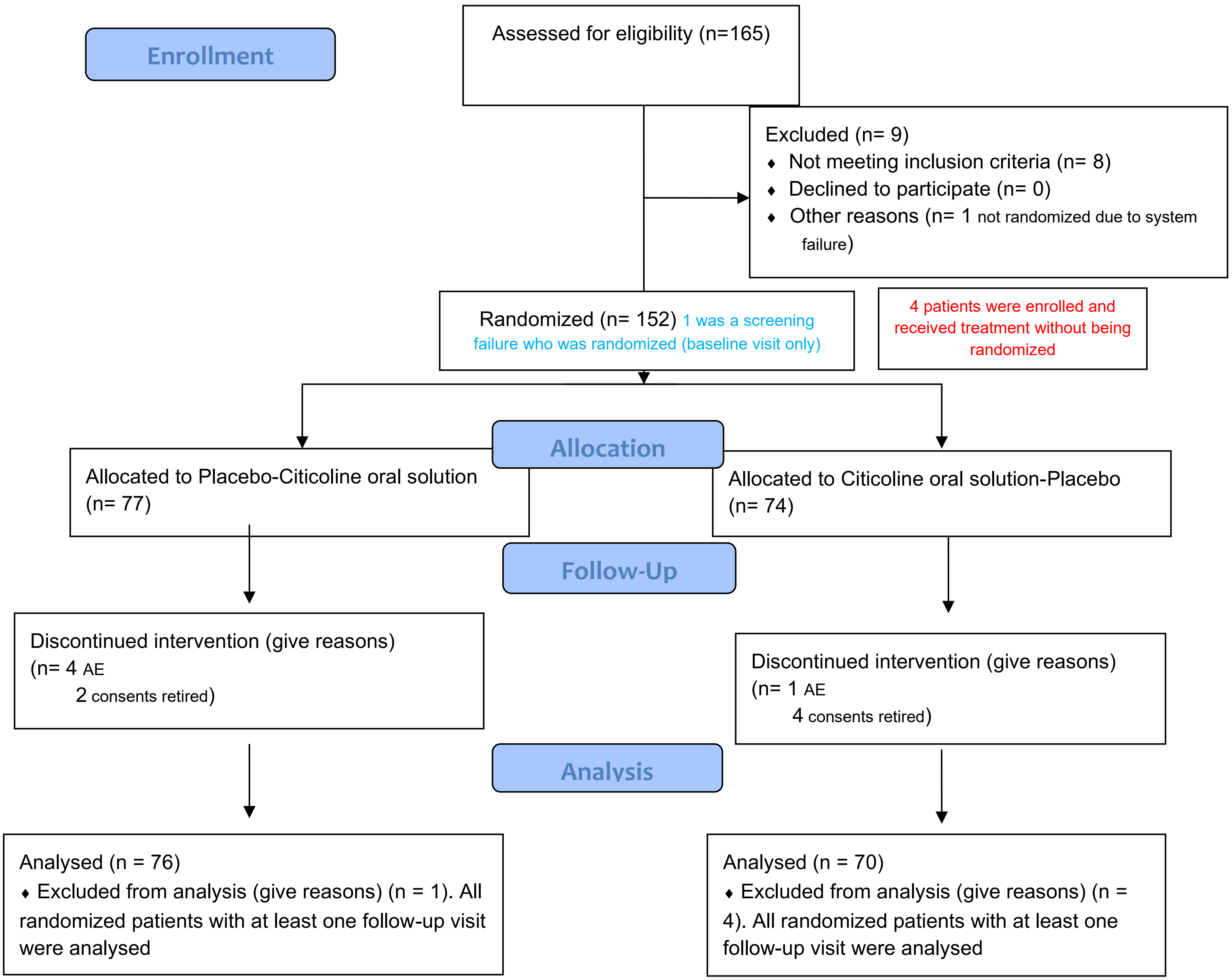

Supplement: Supplementary file 1 — Supplementary file1 (PDF 208 KB) [file 417_2022_5947_MOESM1_ESM.pdf]
